# Protocol optimization for a fast, simple and economical chemical reduction synthesis of antimicrobial silver nanoparticles in non-specialized facilities

**DOI:** 10.1186/s13104-019-4813-z

**Published:** 2019-11-27

**Authors:** Roberto Vazquez-Muñoz, M. Josefina Arellano-Jimenez, Fernando D. Lopez, Jose L. Lopez-Ribot

**Affiliations:** 1 Department of Biology and South Texas Center for Emerging Infectious Diseases, The University of Texas at San Antonio, San Antonio, TX 78249 USA; 2 Department of Physics and Astronomy, The University of Texas at San Antonio, San Antonio, TX 78249 USA

**Keywords:** Silver nanoparticles, Nanoantibiotics, Synthesis method, AgNPs, Metallic nanoparticles

## Abstract

**Objective:**

Silver nanoparticles (AgNPs) can be difficult or expensive to obtain or synthesize for laboratories in resource-limited facilities. The purpose of this work was to optimize a synthesis method for a fast, facile, and cost-effective synthesis of AgNPs with antimicrobial activity, which can be readily implemented in non-specialized facilities and laboratories.

**Results:**

The optimized method uses a rather simple and rapid chemical reduction process that involves the addition of a polyvinylpyrrolidone solution to a warmed silver nitrate solution under constant vigorous stirring, immediately followed by the addition of sodium borohydride. The total synthesis time is less than 15 min. The obtained AgNPs exhibit an aspect ratio close to 1, with an average size of 6.18 ± 5 nm. AgNPs displayed potent antimicrobial activity, with Minimal Inhibitory Concentration values of ≤ 4 µg mL^−1^ for *Staphylococcus aureus* and ≤ 2 µg mL^−1^ for *Candida albicans*. The resulting method is robust and highly reproducible, as demonstrated by the characterization of AgNPs from different rounds of syntheses and their antimicrobial activity.

## Introduction

Silver nanoparticles (AgNPs) are one of the nanomaterials most synthesized worldwide, for a wide diversity of products and applications [1–4]. AgNPs, as nanoantibiotics–nanomaterials with antimicrobial properties-, exert a antiviral [5–7] and broad antimicrobial activity [8–13].

AgNPs are commercially available from diverse companies, most of them USA-based [3], and their cost and obtainability vary due to different factors. Particularly for laboratories in resource-limited countries, AgNPs can be difficult or expensive to obtain. The typical physical presentations of nanoparticles are suspensions or dry-powders. Suspensions are typically low-concentrated, making them unsuitable for antimicrobial assays. Highly concentrated suspensions can be prepared from powders, which are more expensive.

Individual laboratories, mostly in the fields of Physics, Chemistry, and Engineering, synthesize their own AgNPs preparations, often requiring sophisticated and specialized instrumentation. Replicating most of the synthesis methods may pose a challenge for non-dedicated laboratories, due to the cost and difficulties accessing to reagents and equipment. Therefore, the purpose of this work is to optimize a fast, facile, and non-expensive method for synthesizing AgNPs in non-specialized facilities. This easy-to-follow optimization protocol is mostly intended for biological research purposes, sanitation, and nanotechnology education [14, 16]. In the available literature, there are several facile methods for the synthesizing AgNPs [16–19]. This work builds upon this body of literature and aims to develop fast, easy, economical, and highly reproducible methods to obtain easy-to-synthesize antimicrobial nanomaterials without specialized equipment. We posit that the availability of such a method would democratize research on nanomaterials, particularly those non-specialized facilities without access to sophisticated instrumentation, as well as those in resource-limited regions of the world.

## Main text

### Materials and methods

#### Reagents and equipment

Silver nitrate (AgNO_3_), sodium borohydride (NaBH_4_), and Polyvinylpyrrolidone (PVP) from Sigma-Aldrich (MO). Equipment: 200 mL beaker, stirring hot plate, stir bar, thermometer, 1000 µL pipette, 200 µL pipette, 50 mL plastic tube, aluminum foil.

#### AgNPs synthesis

Synthesis via a chemical reduction process, detailed in the Results section.

#### Characterization of AgNPs

UV–Vis spectroscopy. AgNPs surface plasmon was determined by collecting the absorbance profile in a 250–650 nm range wavelength using a UV–Vis-NIR Cary500 (Agilent Technologies). Dynamic Light Scattering (DSL) Analysis. Via a DLS analysis, AgNPs Hydrodynamic size and Zeta potential were assessed, using a Zetasizer Nano ZS (Malvern). High-Resolution Transmission Electron Microscopy (HR-TEM). AgNPs mounted in a Type-B Carbon-coated copper-grids (Ted Pella) were analyzed in a JEOL 2010-F HR-TEM, (accelerating voltage of 200 kV). Shape and size of the AgNPs were defined using the HR-TEM images, whereas the structural lattice was determined using the Selected Area Electron Diffraction (SAED) analysis. Energy Dispersive X-ray spectroscopy (EDS). Elemental characterization was performed using an EDAX EDS detector in the JEOL 2010-F HR-TEM.

#### Microbial strains

The Gram-positive bacterium *Staphylococcus aureus* strain UAMS-1 and the dimorphic yeast *Candida albicans* strain SC5314 were used for these studies. *C. albicans* and *S. aureus* were grown in YPD liquid media and TSB broth, respectively, at 37 °C overnight. Cells from these subcultures were used for the susceptibility tests. A detailed description of the strains preparation and the antimicrobial assays (below) are provided in Additional file 1: Section I.

#### Antimicrobial activity of AgNPs

Antimicrobial susceptibility assays were performed following the Clinical Laboratory Standards Institute (CLSI) M09 and M27 protocols for *S. aureus* and *C. albicans*, respectively [20, 21], with some minor modifications. AgNPs final concentration range from 0.5 to 256 µg mL^−1^. The assays included a control column with untreated cells (with no AgNPs), a control row with only AgNPs (with no cells), and a control blank (only media). Plates were incubated at 37 °C, at 180 rpm, for 24 h for *S. aureus* and at 35 °C for 48 h, for *C. albicans*. Minimal Inhibitory Concentration (MIC) cut-off points were set as the concentration at which no turbidity was visibly observed. In addition, the plates were read spectrophotometrically after the addition of Presto Blue™ Cell Viability Reagent (Invitrogen), as previously described by our group [22]. From the data obtained Dose–response curves were generated and the IC_50_ were calculated. The Minimal Bactericidal Concentration (MBC) and Minimal Fungicidal Concentration (MFC) were also determined. MBC and MFC were set as the lowest concentration of AgNPs that showed fewer than three CFUs. Antimicrobial susceptibility tests were performed using two biological replicates (multi-well plates) with three technical replicates in each plate.

#### Assessment of the stability of AgNPs

AgNPs in liquid suspension were stored at 4 °C in the dark. AgNPs stability over time was determined by measuring the UV–Vis profile and evaluating the antimicrobial activity, every 2 weeks, for 18 weeks, after the initial synthesis. Changes in UV–Vis spectra were estimated analyzing the area under the curve within the wavelength range from 320 to 600 nm, using Prism 6 software (GraphPad^®^).

#### Reproducibility of the synthesis methodology

Once the protocol was fully optimized, several independent rounds of synthesis were performed to demonstrate the robustness and reproducibility of the methodology. UV–Vis spectrophotometry analyses were performed for each round of synthesis. HR-TEM and DLS analysis were performed in two random syntheses, to confirm the presence and traits of the AgNPs. Antimicrobial susceptibility tests were also performed in order to assess the activity of AgNPs from different rounds of synthesis.

### Results and discussion

#### Synthesis of the PVP-AgNPs

The following is a step-by-step description of the synthesis protocol. First, 30 mL of the 15 mM AgNO_3_ stock solution, under vigorous stirring, were warmed at 70 ± 5 °C in a glass beaker on a stirring plate. Second, 5 mL of 30 mM PVP were added to the AgNO_3_ solution, while maintaining under vigorous stirring. Third, immediately after, 300 µL NaBH_4_ were added dropwise to the colorless solution until it turned to a brown or a grayish color (depending on the NaBH_4_ concentration). NaBH_4_ was prepared immediately before it use and was added dropwise. The color change is associated with the AgNPs formation, whereas the turbidity can be used to conjecture if the nanoparticles are highly concentrated. Fourth, the suspension was vigorously stirred for an additional 10 min at 70 ± 5 °C. Finally, the AgNPs were transferred to a light-protected (i.e. wrapped in aluminum foil) Falcon^®^ plastic tube and left to cool down at room temperature, and then stored at 4 °C.

Although there are different methods available for facile syntheses of AgNPs—via the a chemical reduction of the silver ions [16, 18], our method optimizes the synthesis time (< 15 min), the number/preparation of reagents/conditions (i.e. temperature, atmosphere, stabilizers, etc.), and the equipment needed. The protocol was standardized and tested for different concentrations of NaBH_4_, the synthesis was replicated several times to ensure the reproducible formation of AgNPs. Stock solutions were in Milli-Q water: 15 mM AgNO_3_, 3 mM PVP K-10, and NaBH_4_ at different concentrations (4–50 mM). PVP is the coating agent, while NaBH_4_ is the reducing agent. A detailed description of the PVP K-10 and the NaBH_4_ is provided in the Additional file 1: Section II.

AgNPs are synthesized regardless of the concentration range of NaBH_4_ used in this work. Yet, concentration, size, and shape of PVP-AgNPs vary according to the molar ratio of NaBH_4_:Ag. The different synthesis displayed similar antimicrobial activity, as seen in other works [23, 24]. For the subsequent physicochemical characterization described in the following sections, the evaluated PVP-AgNPs were those synthesized with the lowest NaBH_4_ concentration (4 mM). Once the protocol was fully standardized, the synthesis was replicated several times by two different individuals to ensure that the resulting AgNPs obtained from different rounds of synthesis were characterized to confirm that they display similar physical and antimicrobial characteristics in a highly reproducible manner (see below).

#### Characterization of the resulting PVP-AgNPs

##### UV–Vis spectrophotometry

**Fig. 1:**
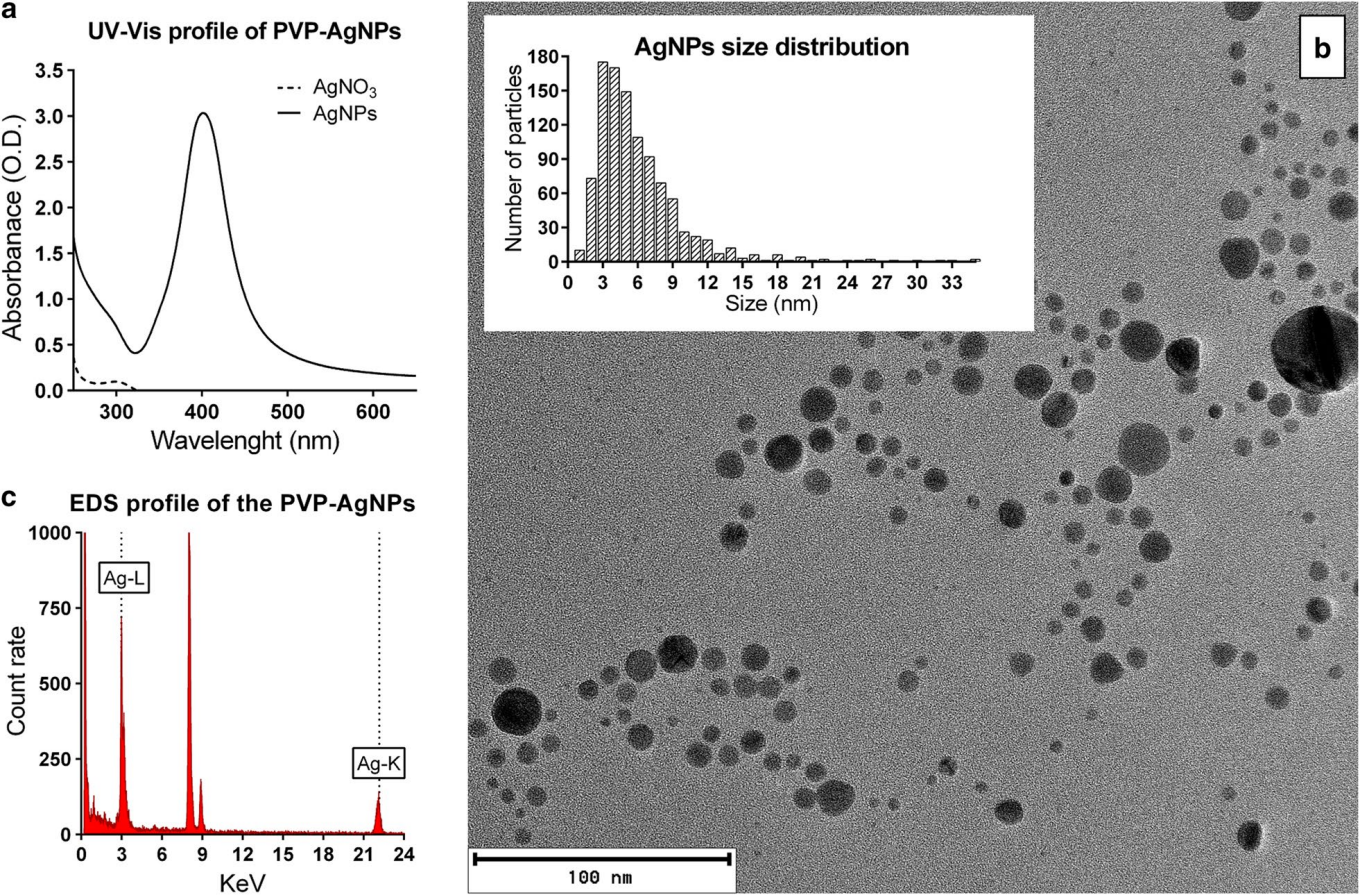
Characterization of silver nanoparticles. **a** UV–Vis profile of AgNPs and AgNO_3_. **b** HR-TEM images show that most of the PVP-AgNPs have an aspect ratio close to 1, with a size range within 2 to 10 nm [n = 1.025 nm]. **c** EDS confirms the presence of silver in the nanoparticles

PVP-AgNPs UV–Vis profile had a single peak with a maximum absorbance from ʎ = 401. The different syntheses showed maximum peaks ranging from 390 to 410 nm, typical for small spheroid PVP-AgNPs. Similarly, the intensity of the absorbance slightly varied in each synthesis, possibly associated with the PVP-AgNPs relative concentration. The AgNO_3_ absorbance profile indicated the conversion to AgNPs. In Fig. 1a shows a representative profile from one synthesis. UV–Vis profiles from different rounds of synthesis are provided in Additional file 1: Fig. S1 [25], demonstrating the high degree of reproducibility of the procedure.

##### HR-TEM analysis

The majority of PVP-AgNPs—from different syntheses—exhibit an aspect ratio close to 1 (Fig. 1b). Other defined shapes were observed in non-significant numbers. PVP-AgNPs average size is 6.18 ± 5 nm, ranging from 1 to 75 nm; > 90% below within 1 to 10 nm (n = 1.025) (Fig. 1b, insert). Few large particles (> 100 nm) and conglomerates were sporadically observed. Additional file 1: Figure S2 [25] shows Images from different syntheses. The EDS chemical composition analysis detected the characteristic X-ray energy bands from silver at 2.984 keV (Lα) and 22.163 keV (Kα) (Fig. 1c).

**Fig. 2:**
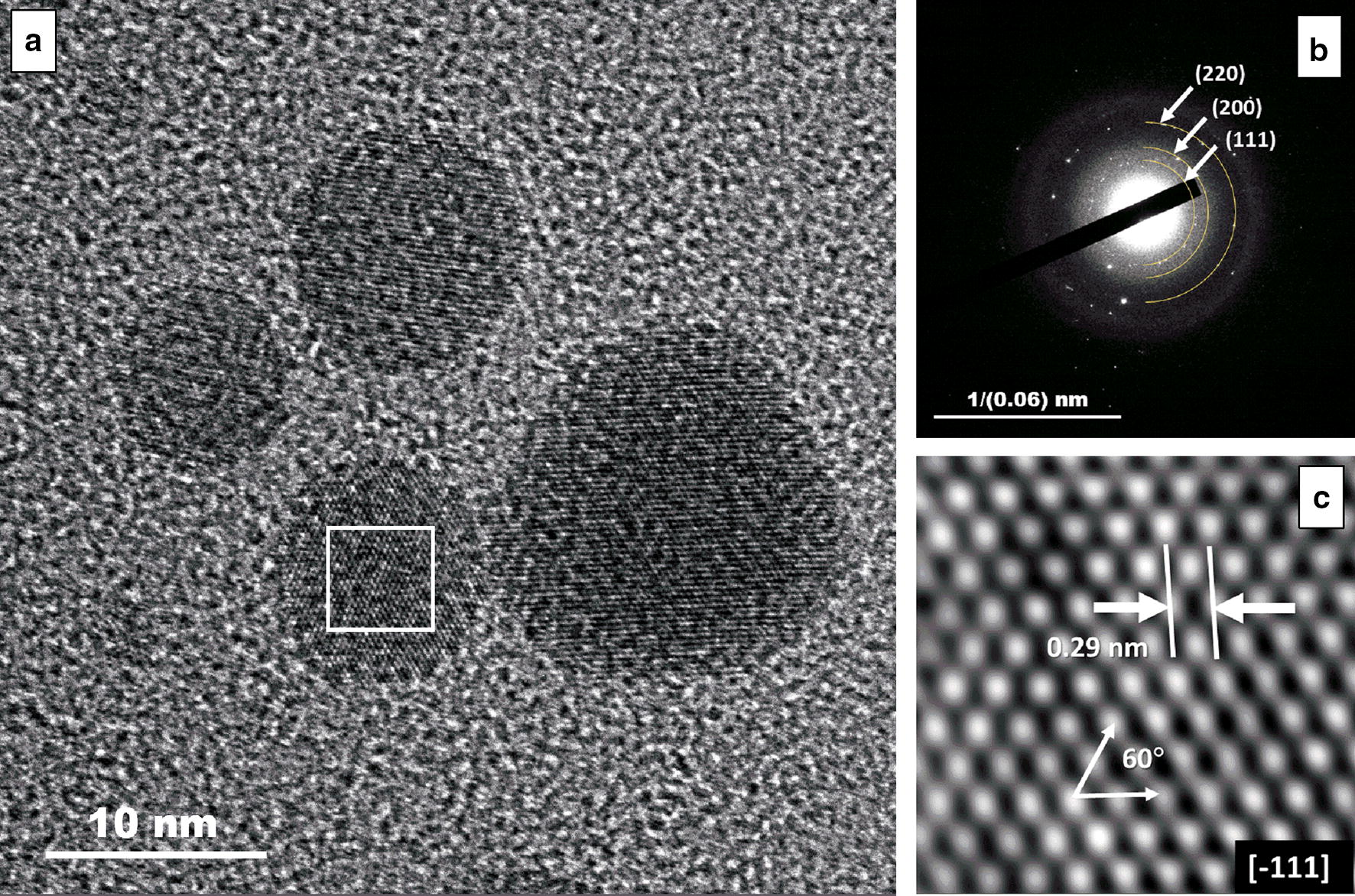
AgNPs Structural lattice. **a** HR-TEM shows that AgNPs are crystalline. **b** The Selected Area Electron Diffraction (SAED) shows that AgNPs the hkl planes {202, 200, 111}. **c** HR-TEM reveals that AgNPs d-spacing is 2.9 Å, with crystalline structure type fcc

HR-TEM unveiled the detailed lattice of PVP-AgNPs (Fig. 2a). The SAED analysis displayed the d-spacing of 2.36, 2.04, and 1.44 nm, corresponding to the hkl planes {202, 200, 111}, respectively (Fig. 2b), according to the standard powder diffraction card of the JCPDS, silver file No. 04–0783. The distance between fringes is 2.9 Å, confirming a face-centered cubic (fcc) crystalline structure (Fig. 2c).

##### Dynamic light scattering

The hydrodynamic size (HS) of the PVP-AgNPs was 35.18 ± 6.55 nm. HS is greater than their metallic core size (35.18 nm and 6.18 nm, respectively), showing that most of the particle volume is in the extended PVP-chains coating. AgNPs Zeta potential is − 16.2 mV (Additional file 1: Figure S3 [25]), revealing a negative surface charge and a low stability score (stability for colloids is achieved at > |30| mV). High Zeta potential values—for more sophisticated syntheses methods—have been reported from − 31 mV [26] to − 45 mV [27]; yet, our Zeta potential is similar to values in other studies, such as − 18.4 mV [28] and + 13.4 [8]. Similarly, Commercial nanoparticles Zeta values are close to ours: + 0.91 mV for Sigma-Aldrich AgNPs (cat. No. 484059) [29], and − 14.1 mV for Vector-Vita PVP-AgNPs [23].

#### Antimicrobial activity of the synthesized AgNPs

**Fig. 3:**
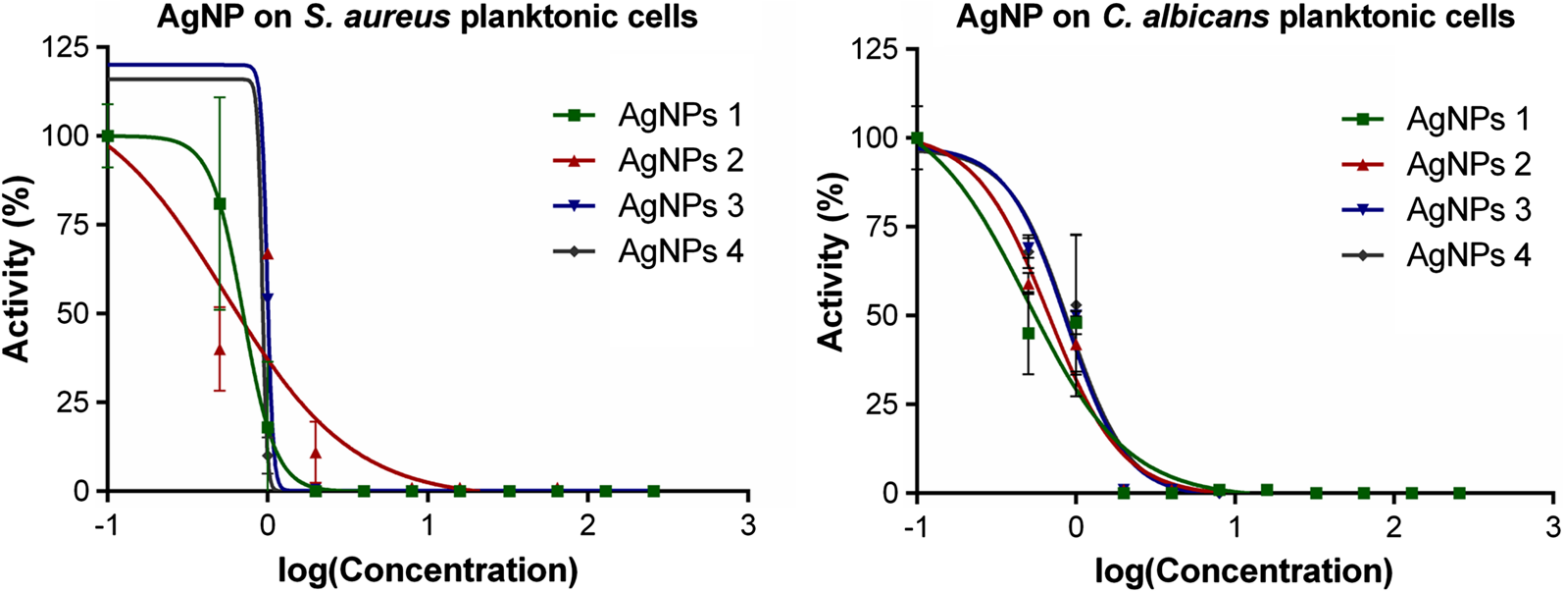
Antimicrobial activity of the AgNPs. Results from dose–response experiments to confirm the inhibitory activity and to determine the potency of AgNPs from different rounds of synthesis against *S. aureus* (left) and against *C. albicans* (right)

The synthesized PVP-AgNPs exhibited potent antimicrobial activity against both *S. aureus* and *C. albicans*. From PVP-AgNPs antimicrobial activity assays for different rounds of synthesis, MIC values were ≤ 4 µg mL^−1^ and ≤ 2 µg mL^−1^ for *S. aureus* and *C. albicans*, respectively; with a representative example shown in Additional file 1: Figure S4 [25]. Figure 3 shows the dose–response curves of antimicrobial activity for AgNPs produced in multiple rounds of synthesis. From these curves, the calculated IC_50_ values ranged from 0.55 to 0.99 µg mL^−1^ for *S. aureus*, whereas for *C. albicans* ranged from 0.49 to 0.90 µg mL^−1^. The MBC for *S. aureus* was ≤ 4 µg mL^−1^, while the MFC for *C. albicans* was ≤ 2 µg mL^−1^, corresponding to their MIC values (Additional file 1: Figure S5 [25]). The antimicrobial activity of our synthesized AgNPs is comparable to values reported in different studies, using similar culture conditions. MIC values in literature range from 0.8 to 13.5 µg mL^−1^ for *S. aureus* (Additional file 1: Table S1) [11, 30], and from 0.4 to 40 µg mL^−1^ for *C. albicans* (Additional file 1: Table S2) [31, 32]. The physicochemical properties of AgNPs depend upon their morphology and capping agent; yet different AgNPs with different traits may display close antimicrobial activity [23].

#### Stability and antimicrobial activity of the synthesized silver nanoparticles after storage

In another series of experiments, we assessed the stability of the resulting AgNPs upon prolonged storage over an extended period of time (up to 18 weeks), by determining changes in their UV–Vis profile and by evaluating their antimicrobial activity every other week. AgNPs surface plasmon changed at week 6, showing a difference of 11.9% when compared to the original profile. Another change in the AgNPs absorbance profile was detected at week 18 (Additional file 1: Figure S6 [25]). Despite these changes, the antimicrobial activity remained unaltered (data not shown).

## Limitations

- Stability and shape mono-dispersity of these PVP-AgNPs is lower than others prepared by more sophisticated methods.
- The PVP-AgNPs suspension contains other silver species, sub-product from the synthesis. These can be removed by washing the PVP-AgNPs.
- AgNPs cytotoxicity on human cells has not yet been evaluated.

## Supplementary information

**Additional file 1: Figure S1.** AgNPs UV–Vis profile. The AgNPs from profile different syntheses displayed a single peak, with a maximum ranging from 390 to 410 nm. **Figure S2.** HR-TEM Analysis. Representative micrographs from different syntheses show that most of the PVP-AgNPs have an aspect ratio close to 1, with an average size lower than 10 nm. Some large particles and agglomerates were also observed sporadically. **Figure S3.** DLS analysis of the AgNPs Zeta potential. The Zeta potential of the AgNPs appears predominantly as a major peak, with a negative charge. The analysis met the system requirements and had a good quality score. **Figure S4.** Photographs of results from susceptibility tests of the AgNPs antimicrobial activity in the multiwall plates. The left panel shows results against *S. aureus* with a MIC value of 2 µg mL^−1^ (indicated by the vertical red line), and the right panel shows results against *C. albicans* with a MIC value of 1 µg mL^−1^. The upper panels show the plates after the incubation with the AgNPs, while the bottom panel shows results using Presto Blue™. **Figure S5.** Microbicidal activity of the AgNPs. The photographs show results of the test for the determination of the AgNPs MBC/MFC values for *S. aureus* (left, 4 µg mL^−1^) and *C. albicans* (right, 2 µg mL^−1^), respectively. Note that these values are highly comparable to the corresponding MIC values. **Figure S6.** AgNPs optical stability over time. The surface plasmon of the silver nanoparticles remained unchanged until week 6, showing a change in the absorbance profile; at week 18 the profile presented another change. **Table S1**. AgNPs antimicrobial activity vs *S. aureus.* **Table S2**. AgNPs antimicrobial activity vs. *C. albicans.*

## Data Availability

Not applicable.

## Abbreviations

AgNO_3_: silver nitrate
AgNPs: silver nanoparticles
CFU: colony-forming units
CLSI: Clinical Laboratory Standards Institute
DSL: dynamic light scattering
EDS: energy dispersive X-ray spectroscopy
HR-TEM: High-Resolution Transmission Electron Microscopy
JCPDS: Joint Committee on Powder Diffraction Standards
MBC: Minimal Bactericidal Concentration
MFC: Minimal Fungicidal Concentration
MIC: Minimal Inhibitory Concentration
NaBH_4_: sodium borohydride
PVP: polyvinylpyrrolidone
SAED: Selected Area Electron Diffraction
