# Supplementary material for: Protocol optimization for a fast, simple and economical chemical reduction synthesis of antimicrobial silver nanoparticles in non-specialized facilities
Source: BMC Res Notes. 2019 Nov 27;12:773. doi: 10.1186/s13104-019-4813-z (PMC6882050; doi:10.1186/s13104-019-4813-z)
Supplement: Supplementary file 1 — Additional file 1: Figure S1. AgNPs UV–Vis profile. The AgNPs from profile different syntheses displayed a single peak, with a maximum ranging from 390 to 410 nm. Figure S2. HR-TEM Analysis. Representative micrographs from different syntheses show that most of the PVP-AgNPs have an aspect ratio close to 1, with an average size lower than 10 nm. Some large particles and agglomerates were also observed sporadically. Figure S3. DLS analysis of the AgNPs Zeta potential. The Zeta potential of the AgNPs appears predominantly as a major peak, with a negative charge. The analysis met the system requirements and had a good quality score. Figure S4. Photographs of results from susceptibility tests of the AgNPs antimicrobial activity in the multiwall plates. The left panel shows results against S. aureus with a MIC value of 2 µg mL−1 (indicated by the vertical red line), and the right panel shows results against C. albicans with a MIC value of 1 µg mL−1. The upper panels show the plates after the incubation with the AgNPs, while the bottom panel shows results using Presto Blue™. Figure S5. Microbicidal activity of the AgNPs. The photographs show results of the test for the determination of the AgNPs MBC/MFC values for S. aureus (left, 4 µg mL−1) and C. albicans (right, 2 µg mL−1), respectively. Note that these values are highly comparable to the corresponding MIC values. Figure S6. AgNPs optical stability over time. The surface plasmon of the silver nanoparticles remained unchanged until week 6, showing a change in the absorbance profile; at week 18 the profile presented another change. Table S1. AgNPs antimicrobial activity vs S. aureus. Table S2. AgNPs antimicrobial activity vs. C. albicans. [file 13104_2019_4813_MOESM1_ESM.docx]

**Protocol optimization for a fast, simple and economical chemical reduction synthesis of antimicrobial silver nanoparticles in non-specialized facilities**

**Additional file**

**I. Notes on the microbial strains and the antimicrobial assays**.

1. Microbial strains.

The Gram-positive bacterium *Staphylococcus aureus* strain UAMS-1 and the dimorphic yeast *Candida albicans* strain SC5314 were used for these studies. *S. aureus* is a leading cause of hospital bacterial infections (30) while *C. albicans* is an opportunistic pathogenic fungus and the main causative agent of candidiasis, affecting an increasing number of immunosuppressed and medically compromised patients, with unacceptably high mortality rates (31,32). Frozen stocks of *S. aureus* strain UAMS-1 were subcultured onto tryptic soy agar (TSA) plates (BD Difco, MD) at 37 °C for 24 h, then they were subcultured in 10 ml of tryptic soy broth (TSB) (BD Difco, MD) in an orbital shaker at 37°C overnight. Frozen *C. albicans* strain SC5314, stored at −80°C in glycerol stock, was cultured on yeast extract-peptone-dextrose (YPD) agar plates (BD Difco, MD) and incubated at 35 °C for 48 h. A loopful of *S. aureus* cells from the TSB agar plates and of the *C. albicans* cells from the YPD plates were inoculated, into 20 ml of YPD liquid media, and in TSB broth, respectively, and grown in an orbital shaker at 37°C overnight. Cells from these bacterial and fungal subcultures were used to prepare the initial inoculum for susceptibility tests.

1. Antimicrobial assays.

Briefly, an initial inoculum of *S. aureus* was adjusted to 10^6^ cells mL^-1^ in Mueller Hinton (MH) broth, whereas *C. albicans* initial inoculum was prepared by adjusting to 10^3^ cells mL^-1^ in RPMI 1640 culture media (Corning®; Cellgro®, VA) with 165 mM morpholinepropanesulfonic acid (MOPS, Thermo-Fisher Scientific, Waltham, MA) at pH 6.9. Then, 50 µL of the initial inocula were added to wells of 96 well microtiter plates. AgNPs were prepared in a two-fold dilution series in either MH (for *S. aureus*) or RPMI (for *C. albicans*) media. Immediately, 50 µL of the AgNPs two-fold dilution series were added to the multi-well plates containing the microbial inocula. From the Dose-Response curves, the IC_50_ values, defined as the concentration of drug required to reduce growth by 50%, were determined by fitting the normalized results to the variable slope Hill equation (for determining the nonlinear drug dose-response relationship) using Prism 8 (GraphPad Software Inc).

To determine the Minimal Bactericidal Concentration (MBC) and Minimal Fungicidal Concentration (MFC), we followed the following procedure: after reading the plates for establishing the MICs, 10 μl from each well containing the different dilutions of AgNPs as well as control wells, were subcultured, in TSA and YPD agar plates for *S. aureus* a *C. albicans*, respectively; and these plates were incubated for 24 h at 37°C.

**II. Notes on the AgNPs synthesis protocol**.

For the proposed synthesis, the stock solutions were prepared using Milli-Q water, although distilled water can be used too. Yet, it will impact the quality of the synthesized AgNPs. Still, the AgNPs will exhibit antimicrobial activity.

For the synthesis, we selected the PVP K-10 and the NaBH_4_. PVP is a non-toxic polymer widely used in medical products and cosmetics. When used for nanoparticle synthesis, PVP grants stability (1), biocompatibility (2,3), and also acts as a dispersant (4). PVP coating confers a negative surface charge to the AgNPs and has no antimicrobial properties by itself. The charge influences the AgNPs – cell interaction (5,6). Also, the capping influences the AgNPs antimicrobial properties (7). PVP comes in different sizes, and the size and concentration of this polymer influences in the AgNPs overall size and shape, for the purposes of this work we selected the PVP K-10, which was the smallest available (M.W.=10,000 Daltons). Sodium borohydride (NaBH_4_) is a good reducing agent, relatively inexpensive and easily handled. NaBH_4_ rapidly reduces the silver ion to zero-valent silver, leading to a formation of nanostructures under the proper conditions. Moreover, NaBH_4_ has no inhibitory activity against the microbial cells at the tested concentrations.

Important. NaBH_4_ it loses its activity fast when diluted in water, thus it has been used as soon after it is prepared. During the synthesis, it has to be added slowly since it induces an exothermic reaction that can generate a lot of heat and increase the temperature of the solution.

**III. AgNPs characterization**.

1. UV Vis spectrophotometry profile of the PVP-AgNPs from different syntheses demonstrates that the method is highly reproducible

AgNPs UV-Vis profile from the different syntheses displayed a single peak, with a maximum peak ranging from 390 to 410 nm. The intensity of the absorbance in each synthesis was slightly different (**Fig. S1**).





**Figure S1: AgNPs UV-Vis profile**. The AgNPs from profile different syntheses displayed a single peak, with a maximum ranging from 390 to 410 nm. https://doi.org/10.6084/m9.figshare.10248890

1. HR-TEM images for the AgNPs

Most of the AgNPs from the different syntheses have an average size lower than 10 and have an aspect ratio close to 1 (**Fig. S2**). Few large particles (>100 nm) and conglomerates were sporadically observed.







**Figure S2: HR-TEM Analysis.** Representative micrographs from different syntheses show that most of the PVP-AgNPs have an aspect ratio close to 1, with an average size lower than 10 nm. Some large particles and agglomerates were also observed sporadically. <https://doi.org/10.6084/m9.figshare.10248887>

1. Zeta potential of the AgNPs

The Zeta potential analysis revealed that AgNPs synthesized using the optimized protocol have a negative surface charge and a stability score lower |30| mV (**Fig. S3**).


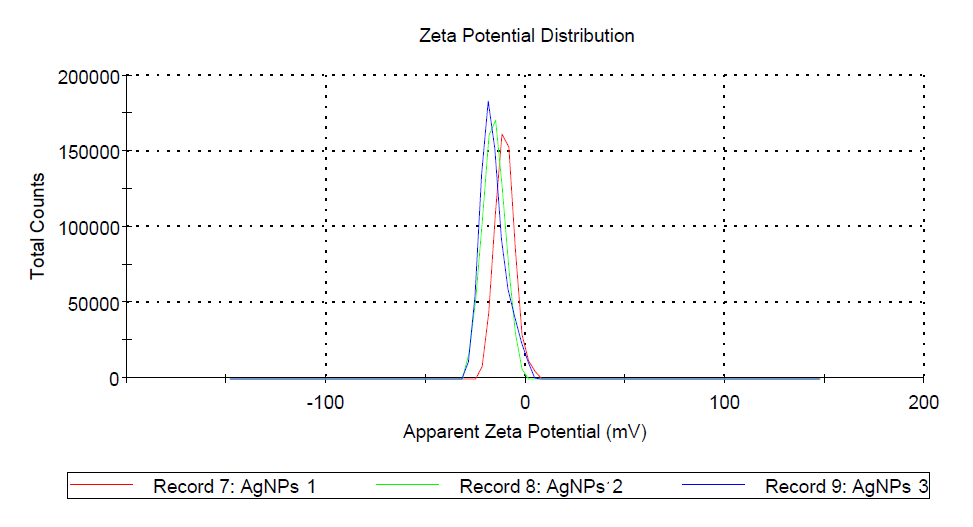


**Figure S3: DLS analysis of the AgNPs Zeta potential**. The Zeta potential of the AgNPs appears predominantly as a major peak, with a negative charge. The analysis met the system requirements and had a good quality score. <https://doi.org/10.6084/m9.figshare.10248893>

**IV. Antimicrobial activity of AgNPs**.

1. AgNPs Antimicrobial activity.

Antimicrobial activity on *S. aureus* and *C. albicans* planktonic cells.


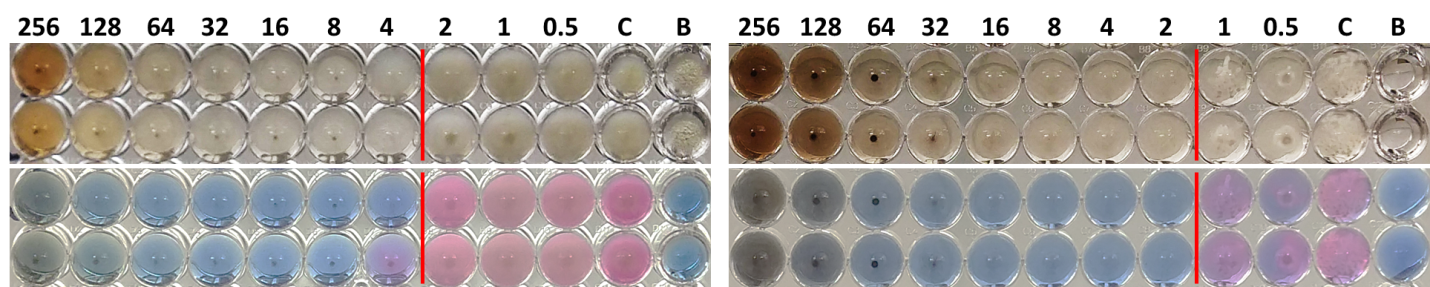


**Figure S4: Photographs of results from susceptibility tests of the AgNPs antimicrobial activity in the multiwall plates.** The left panel shows results against *S. aureus* with a MIC value of 2 µg mL^-1^ (indicated by the vertical red line), and the right panel shows results against *C. albicans* with a MIC value of 1 µg mL^-1^. The upper panels show the plates after the incubation with the AgNPs, while the bottom panel shows results using Presto Blue^TM^. <https://doi.org/10.6084/m9.figshare.10248896>


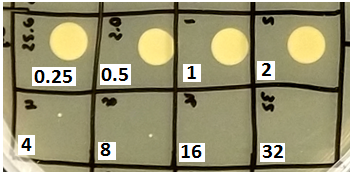

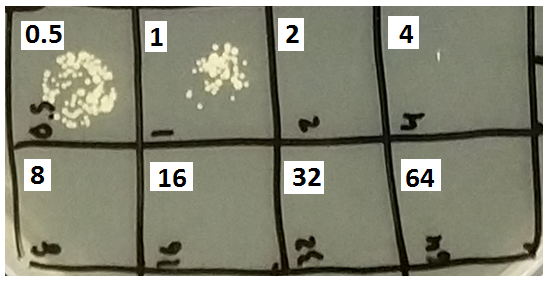


**Figure S5: Microbicidal activity of the AgNPs**. The photographs show results of the test for the determination of the AgNPs MBC/MFC values for *S. aureus* (left, 4 µg mL^-1^ ) and *C. albicans* (right, 2 µg mL^-1^), respectively. Note that these values are highly comparable to the corresponding MIC values. <https://doi.org/10.6084/m9.figshare.10248899>

1. Antimicrobial activity of AgNPs in the literature.

The antimicrobial activity of our synthesized PVP-AgNPs is comparable to other reported in different studies, using similar culture conditions. MIC values in the literature range from 0.8 µg mL^-1^ to 13.5 µg mL^-1^ for *S. aureus* (**Table S1**), and from 0.4 µg mL^-1^ to 40 µg mL^-1^ for *C. albicans* (**Table S2**).

| **Table S1**. AgNPs antimicrobial activity vs *S. aureus* | | |
| --- | --- | --- |
| AgNPs  characteristics | MIC  (µg ml^-1^) | Ref. |
| **6.18 ± 5 nm, spheroids. Stabilizer: PVP** | **4** | **This work** |
| 13 ±3 nm, Polyhedral. Stabilizer: Tannic acid | 1.56 | (8) |
| 16-25 nm, shape not mentioned. Stabilizer: N/A | 0.8 | (9) |
| 20 nm, spherical, biosynthesis | 20 | (10) |
| 20 nm, ellipsoid. Stabilizer: PVP  7 nm, ellipsoid. Stabilizer: PEG | 10.1  13.5 | (11) |
| 1 nm, Spheroids, Uncapped | 12.5 | (12) |
| 9 nm, shape not mentioned. Stabilizer: PVA. | 1.95 | (13) |
| 8.6 nm, poly-shaped. Stabilizer: N-Cholyl L-Valine. | 3.5 | (14) |
| 18.34 nm, spheroids & polyhedrons. Stabilizer: N/A | 2 | (15) |
| 5-10 nm, shape not mentioned. Stabilizer: N/A. From Nanoparticle Biochem, Inc. | 12.5 | (16) |
| Culture conditions are based on the CLSI’s A07 protocols for *S. aureus*: 10^6^ cells mL^-1^, Mueller Hinton broth, 24 h at 37 ^o^C. | | |

| **Table S2**. AgNPs antimicrobial activity vs. *C. albicans* | | |
| --- | --- | --- |
| AgNPs  characteristics | MIC  (µg mL^-1^) | Ref. |
| **6.18 ± 5 nm, spheroids. Stabilizer: PVP** | **2** | **This work** |
| 5 nm, shape polyhedron-like. Stabilizer: NH3.  5 nm, shape polyhedron-like. Stabilizer: PVP | 0.4  0.8 | (17) |
| 8.6 nm, poly-shaped. Stabilizer: N-Cholyl amino acids. | 1.75 | (14) |
| 21.6 nm, spherical. Stabilizer: N/A | 40 | (18) |
| Culture conditions are based on the CLSI’s M27 protocol for *C.* *albicans*, 10^3^ cells mL^-1^, RPMI media, and 48 h, at 35 ^o^C. | | |

**V. Stability of AgNPs over time**.





**Figure S6: AgNPs optical stability over time**. The surface plasmon of the silver nanoparticles remained unchanged until week 6, showing a change in the absorbance profile; at week 18 the profile presented another change. <https://doi.org/10.6084/m9.figshare.10248902>

**References**

1. Tejamaya M, Römer I, Merrifield RC, Lead JR. Stability of citrate, PVP, and PEG coated silver nanoparticles in ecotoxicology media. Environ Sci Technol. 2012;46(13):7011–7.

2. Lyutakov O, Kalachyova Y, Solovyev A, Vytykacova S, Svanda J, Siegel J, et al. One-step preparation of antimicrobial silver nanoparticles in polymer matrix. J Nanoparticle Res [Internet]. 2015 Mar 6 [cited 2019 Jul 12];17(3):120. Available from: http://link.springer.com/10.1007/s11051-015-2935-3

3. Rogero SO, Malmonge SM, Lugão AB, Ikeda TI, Miyamaru L, Cruz ÁS. Biocompatibility Study of Polymeric Biomaterials. Artif Organs [Internet]. 2003 May 9 [cited 2019 Jul 12];27(5):424–7. Available from: http://doi.wiley.com/10.1046/j.1525-1594.2003.07249.x

4. Koczkur KM, Mourdikoudis S, Polavarapu L, Skrabalak SE. Polyvinylpyrrolidone (PVP) in nanoparticle synthesis. Dalt Trans [Internet]. 2015 Oct 13 [cited 2019 Jul 15];44(41):17883–905. Available from: http://xlink.rsc.org/?DOI=C5DT02964C

5. Abbaszadegan A, Ghahramani Y, Gholami A, Hemmateenejad B, Dorostkar S, Nabavizadeh M, et al. The Effect of Charge at the Surface of Silver Nanoparticles on Antimicrobial Activity against Gram-Positive and Gram-Negative Bacteria: A Preliminary Study. 2015 [cited 2019 Jul 15]; Available from: http://dx.doi.org/10.1155/2015/720654

6. El Badawy AM, Silva RG, Morris B, Scheckel KG, Suidan MT, Tolaymat TM. Surface Charge-Dependent Toxicity of Silver Nanoparticles. Environ Sci Technol [Internet]. 2011 Jan [cited 2019 Jul 15];45(1):283–7. Available from: https://pubs.acs.org/doi/10.1021/es1034188

7. Gnanadhas DP, Ben Thomas M, Thomas R, Raichur AM, Chakravortty D. Interaction of silver nanoparticles with serum proteins affects their antimicrobial activity in vivo. Antimicrob Agents Chemother [Internet]. 2013 Oct 1 [cited 2019 Mar 6];57(10):4945–55. Available from: http://www.ncbi.nlm.nih.gov/pubmed/23877702

8. Orlowski P, Zmigrodzka M, Tomaszewska E, Ranoszek-Soliwoda K, Czupryn M, Antos-Bielska M, et al. Tannic acid-modified silver nanoparticles for wound healing: the importance of size. Int J Nanomedicine [Internet]. 2018 [cited 2019 Apr 6];13:991–1007. Available from: http://www.ncbi.nlm.nih.gov/pubmed/29497293

9. Esmaeillou M, Zarrini G, Ahangarzadeh Rezaee M, Shahbazi Mojarrad J, Bahadori A. Vancomycin Capped with Silver Nanoparticles as an Antibacterial Agent against Multi-Drug Resistance Bacteria. Adv Pharm Bull [Internet]. 2017 Sep [cited 2019 Apr 6];7(3):479–83. Available from: http://www.ncbi.nlm.nih.gov/pubmed/29071232

10. Hoskote Anand KK, Mandal BK. Activity study of biogenic spherical silver nanoparticles towards microbes and oxidants. Spectrochim Acta - Part A Mol Biomol Spectrosc [Internet]. 2015;135:639–45. Available from: http://dx.doi.org/10.1016/j.saa.2014.07.013

11. Suchomel P, Kvitek L, Panacek A, Prucek R, Hrbac J, Vecerova R, et al. Comparative Study of Antimicrobial Activity of AgBr and Ag Nanoparticles (NPs). PLoS One. 2015;10(3):1–16.

12. Romero-Urbina DG, Lara HH, Velázquez-Salazar JJ, Arellano-Jiménez MJ, Larios E, Srinivasan A, et al. Ultrastructural changes in methicillin-resistant Staphylococcus aureus induced by positively charged silver nanoparticles. Beilstein J Nanotechnol [Internet]. 2015 Dec 15;6:2396–405. Available from: https://www.beilstein-journals.org/bjnano/articles/6/246

13. Wady AF, Machado AL, Foggi CC, Zamperini CA, Zucolotto V, Moffa EB, et al. Effect of a Silver Nanoparticles Solution on *Staphylococcus aureus* and *Candida* spp. J Nanomater [Internet]. 2014 Aug 20 [cited 2019 Apr 17];2014:1–7. Available from: http://www.hindawi.com/journals/jnm/2014/545279/

14. Annadhasan M, SankarBabu VR, Naresh R, Umamaheswari K, Rajendiran N. A sunlight-induced rapid synthesis of silver nanoparticles using sodium salt of N-cholyl amino acids and its antimicrobial applications. Colloids Surfaces B Biointerfaces [Internet]. 2012 Aug 1 [cited 2019 Apr 6];96:14–21. Available from: https://www.sciencedirect.com/science/article/pii/S0927776512001798

15. Mirzajani F, Ghassempour A, Aliahmadi A, Esmaeili MA. Antibacterial effect of silver nanoparticles on Staphylococcus aureus. Res Microbiol [Internet]. 2011 Jun 1 [cited 2018 May 18];162(5):542–9. Available from: https://www.sciencedirect.com/science/article/pii/S0923250811000829?via%3Dihub

16. Ma A, Hm K, Aa K, Malik A, Sultan A, Shahid M, et al. Evaluation of antibacterial activity of silver nanoparticles against MSSA and MRSA on isolates from skin infections. Res Artic Biol Med. 2011;3(2):141–6.

17. Monteiro DR, Silva S, Negri M, Gorup LF, De Camargo ER, Oliveira R, et al. Silver nanoparticles: Influence of stabilizing agent and diameter on antifungal activity against Candida albicans and Candida glabrata biofilms. Lett Appl Microbiol. 2012;54(5):383–91.

18. Radhakrishnan VS, Dwivedi SP, Siddiqui MH, Prasad T. In vitro studies on oxidative stress-independent, Ag nanoparticles-induced cell toxicity of Candida albicans, an opportunistic pathogen. Int J Nanomedicine [Internet]. 2018 [cited 2019 May 6];13(T-NANO 2014 Abstracts):91–6. Available from: http://www.ncbi.nlm.nih.gov/pubmed/29593404
